# Supplementary material for: Overexpression of ThMYC4E Enhances Anthocyanin Biosynthesis in Common Wheat
Source: Int J Mol Sci. 2019 Dec 24;21(1):137. doi: 10.3390/ijms21010137 (PMC6982250; doi:10.3390/ijms21010137)
Supplement: Supplementary file 1 [file ijms-21-00137-s001.pdf]

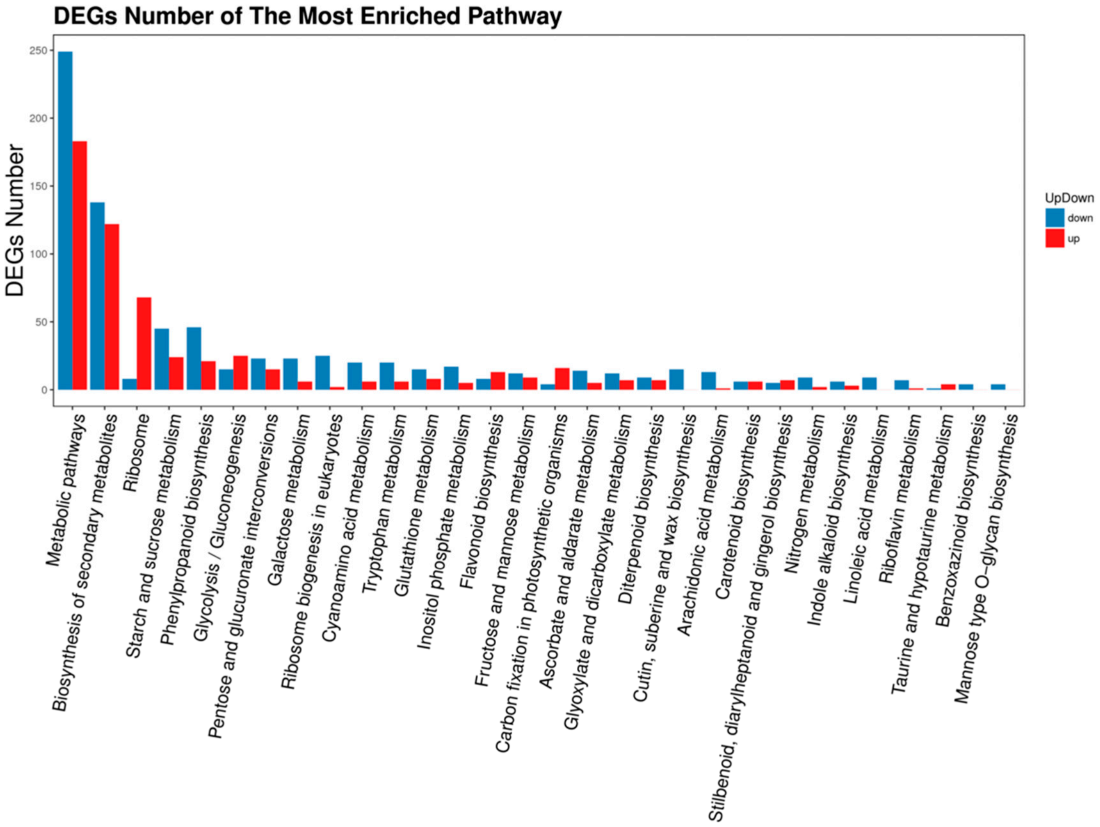

Figure S1. The number of differential expressed unigenes in KEGG pathway. X axis represent pathway name. Y axis represent the number of differential expressed genes (DEGs).Blue columns represent the number of up-regulated genes, and red columns represents number of the down-regulated genes.

Table S1. Oligo nucleotide primers used in this work.

Name	Forward (5'-3')	Reverse (5'-3')
ThMYC4E-pLGY02	GGGGTACCATGCGGGAAATAGCTAC	GGACTAGTCTATATAGCTTTCTGAA
ThMYC4E	GCCGTCGGGGAAGCAATTCGG	CCCAGGTGCCACGATAGCAGAAC
hyg	GTCTCCGACCTGATGCAGCTCTCGG	GTCCGTCAGGACATTGTTGGAG
CHS-RT	TGGAAAACAACCTACTACATACAGC	CAGACTAACAGGAGATCGAATGCAT
CHI-RT	CGTCCTCGTATTTGTCCGCTG	GAACCATAGTCACATATCACGAGG
F3H-RT	TGATTGATGCGTGGTGGGA	CAGAAACCAGTACGAAATATACGC
F3'5'H-RT	GGGCTAGGATCAGATCAACTCG	AATACAAAACAGTACGCACAGAAAAC
DFR-RT	GCTCATCACAGGGAATGAAGC	GTCGTCGACGCCTGCGA
ThMYC4E-RT	CATGGACGAGCCCTTTGAGT	CTCGGATGATCCCTTCCACG
Rc-D1-RT	GAACATCAAGCGGGGCAACATC	GCGTGCTGTTCCAGTAGTTCTT
Tubulin-RT	CAAGGAGGTGGACGAGCAGATG	GACTTGACGTTGTTGGGGATCCA
